# Realization of structural transformation for the enhancement of magnetic and magneto capacitance effect in BiFeO_3_–CoFe_2_O_4_ ceramics for energy storage application

**DOI:** 10.1038/s41598-021-81867-4

**Published:** 2021-01-26

**Authors:** Muniyandi Muneeswaran, Ali Akbari-Fakhrabadi, Miguel Angel Gracia-Pinilla, Juliano C. Denardin, Nambi Venkatesan Giridharan

**Affiliations:** 1 Advanced Materials Laboratory, Department of Mechanical Engineering, University of Chile, Santiago, Chile; 2 Universidad Autónoma de Nuevo León, Faculated de Ciencias Fisico-Matemáticas, Av. Universidad, Cd. Universitian, C.P. 66450 San Nicolás de los Graza, NL Mexico; 3 Department of Physics, CEDENNA, University of Santiago, 7190006 Santiago, Chile; 4 Advanced Functional Materials Laboratory, Department of Physics, National Institute of Technology, Tiruchirappalli, 620015 India

**Keywords:** Energy science and technology, Materials science, Physics

## Abstract

In this study, (1 − x) BFO-xCFO (CFO, x = 0.00, 0.05, 0.10 and 0.30) ceramics were synthesized by a solid-state reaction method; their compositions were driven by structural, microstructural, vibrational, electrical, magnetic properties; their enhanced magneto capacitance (MC) effect have also been carried out. Reitveld refinement studies of X-ray diffraction data shows composition-driven structural phase transformation from rhombohedral (*R3c*) to tetragonal (*P4mm*). Two phonon scattering Raman modes were observed for the higher wavenumber which supports the crystal structural transition in the BFO-CFO. Ferroelectric polarization shows that the polarization increased with increasing CFO concentration, which describes the changes of the polar symmetry of the crystal structure from rhombohedral (*R3c*) to tetragonal (*P4mm*). In Further, the maximum efficiency of energy density (η = 68.65%), reversible energy density of 0.138 J/cm^3^ and the strong magneto capacitance was observed in 0.9BFO-0.1CFO, which belongs to the morphotropic phase boundary (MPB) region near to the BiFeO_3_-rich region. The magnetic response analysis has shown, the saturation magnetization (M_s_) values of 83 emu/gm and 139 emu/gm for pure CFO and 0.7BFO-0.3CFO composite, respectively, and their magnetic behaviours were also confirmed with Arrott–Belov–Kouvel (ABK) plot.

## Introduction

In recent years, multiferroic materials has been increasingly researched as electric polarization which can be controlled by magnetic field and the order reversed due to the existence of coupling between the ferroic orders^1^. Multiferroic materials which have exhibited more than one ferroic orders such as ferromagnetism, ferroelectricity and ferroelasticity has drawn attention widely in the field of solid-state physics and in numerous technological fields for developing new potential applications such as low power consumption and electric field controlled spintronic devices, electrically assisted magnetic recording, multi-state memory, magnetoresistance and reversible electrical switching of spin polarization etc. Cross-coupling between the magnetic and electrical orders termed as magnetoelectric (ME) coupling is specifically fascinating^2–4^ as this coupling allows electrical polarization controlled by a magnetic field, vice versa, the manipulation of magnetization by varying an electrical field^5–7^.

BiFeO_3_ (BFO) with a weak magnetism at room temperature is one of the most promising multiferroic materials with the giant ferroelectric polarization of 100 µC/cm^2^ and a very high Curie temperature (T_C_ = 1103 K) and Néel temperature (T_N_ = 643 K)^8^. The origin of its ferroelectricity is from the stereochemical activity of ‘Bi’ 6s^2^ lone pair electron and the reason for inducing magnetic ordering is due to the partially filled ‘d’ shells. It has exhibited a single phase of rhombohedrally distorted perovskite structure with *R3c* space group at room temperature. This distorted perovskite structure with FeO_6_ octahedra paves way for coupling of magnetism and ferroelectricity^9^. However, pure BFO is limited for the enhancement of ME coupling. It possesses high leakage current by oxygen vacancy, high dielectric loss, weak antiferromagnetic order, and structural instability due to the formation of impurities phases like Bi_2_Fe_4_O_9_ and Bi_25_Fe_2_O_39_ because of the kinetics of formation. These drawbacks can be overcome by substituting suitable rare elements at Bi site, transition elements at Fe site and co-doping with both sites of BFO to make composite system. Recent studies on BFO–CFO composite have shown that the composite system play an important role in modifying the crystal structure which leads to enhance the electrical, magnetic, and ME coupling properties. Khalid et al.^10^ successfully prepared (1 − x)BiFe_0.95_Mn_0.05_O_3_–xCoFe_2_O_4_ (0.2 ≤ x ≤ 0.7) core–shell nano-composites and observed structural transformation from rhombohedral (*R3c*) to rhombohedrally distorted cubic-type perovskite (*R3m*) in 0.8BFMO-0.2CFO which gives maximum polarization, highest efficiency of energy density and enhanced the magneto capacitance indicating that these composites have a potential for application in energy storage devices.

The enhancement of ferromagnetism and ferroelectricity of BFO–CFO system have been ascribed to several factors including contributions of valence fluctuations of the Fe ion^11,12^. If Fe^3+^ is reduced to Fe^2+^, the system can then compensate ionically by forming oxygen vacancies (V_O_^••^) or electronically with holes (h•). These reactions are thus described in Kröger-Vink notation:

$${\text{2Fe}}^{{\text{x}}}_{{{\text{Fe}}}} + {\text{ O}}_{{\text{o}}}^{{\text{x}}} \to {\text{ 2Fe}}\prime_{{{\text{Fe}}}} + {\text{ V}}_{{\text{o}}}^{ \bullet \bullet } + \, \raise.5ex\hbox{$\scriptstyle 1$}\kern-.1em/ \kern-.15em\lower.25ex\hbox{$\scriptstyle 2$} {\text{ O}}_{{{2}({\text{g}})}}$$

$${\text{Fe}}^{{\text{x}}}_{{{\text{Fe}}}} \to {\text{ Fe}}\prime_{{{\text{Fe}}}} + {\text{ h}}^{ \bullet }$$

It has been generating the electronic charge carriers that contribute to leakage current density or oxygen vacancy in the BFO. The results suggest that the presence of secondary phases like Bi_2_Fe_4_O_9_ and Bi_24_Fe_2_O_39_ has been known to affect the electrical properties.

On substituting Co^3+^ and Fe^3+^ ions, point defects with an effective negative charge is created. These defects are equalized by defects of effective positive charge intending to conserve overall charge neutrally which may possibly be electron holes or oxygen vacancies. By using Kröger-Vink notation, these two possible substitutions can be written as:

$${\text{Co}}_{{3}} {\text{O}}_{{4}} \to {\text{ 3Co}}^{{\text{x}}}_{{{\text{Co}}}} + {\text{ 3O}}_{{\text{o}}}^{{\text{x}}} + {\text{ V}}_{{\text{o}}}^{ \bullet \bullet } + \, \raise.5ex\hbox{$\scriptstyle 1$}\kern-.1em/ \kern-.15em\lower.25ex\hbox{$\scriptstyle 2$} {\text{ O}}_{{2}} \left( {\text{g}} \right)$$

$${\text{Co}}_{{3}} {\text{O}}_{{4}} + {\text{Fe}}_{{2}} {\text{O}}_{{3}} \to {\text{ 3Co}}^{{\text{x}}}_{{{\text{Fe}}}} + {\text{ 2Fe}}^{{\text{x}}}_{{{\text{Fe}}}} + {\text{ 6O}}_{{\text{o}}}^{{\text{x}}} + {\text{ V}}_{{\text{o}}}^{ \bullet \bullet } + \, \raise.5ex\hbox{$\scriptstyle 1$}\kern-.1em/ \kern-.15em\lower.25ex\hbox{$\scriptstyle 2$} {\text{ O}}_{{2}} \left( {\text{g}} \right)$$

These lattice defect change will affect the electrical and magnetic properties. For this reason, CFO has been chosen for making BFO-CFO composites at different molar concentrations as it exhibits high magnetic response, coercivity, and magneto crystalline anisotropy. It also has potential applications in data storage devices, ferrofluid technology, magnetic resonance imaging, optoelectronics, and in targeted drug delivery^13,14^. Thus, CFO is assumed to be a suitable ferrite in designing composites with BFO to improve the ME coupling. In addition to that CFO has a high magnetic nature which is expected to enhance the magnetic response of the composites.

In this work, a systematic study on the structural phase transformation in BFO-CFO ceramics has been made by varying the CFO concentration in order to investigate the first-time peculiarities of multiferroic behaviour has been done and their compositional driven microstructural, electrical, magnetic and energy storage density properties and enhanced magneto capacitance effect have also been carried out.

## Experimental details

A series of (1 − x) BFO-xCFO (CFO, x = 0.00, 0.05, 0.10 and 0.30) ceramics along with pure BFO and CFO powders were synthesized by the conventional solid-state reaction method using high purity analytical grade of Bi_2_O_3_, Fe_2_O_3_ and Co_3_O_4_ reagents (Sigma Aldrich) as starting materials and the stoichiometric proportions of precursor reagents were calculated for each sample and well mixed and ground with agate mortar. These powders were calcinated at 840 °C for 3 h. The calcined ceramic powders were uniaxially pressed (90 MPa) to fabricate into pellets with 8 mm diameter and 0.90 mm thickness. Then, the prepared pellets were sintered at 980 °C for 5 h.

The crystal structures of powders were identified with X-ray diffraction (XRD) analysis by using a Philips X′Pert/MPD diffraction system (CuΚ_α_ radiation, λ = 1.5406 Å) and their phase fractions and crystal structure information were calculated by Rietveld refinement using General Structure Analysis System (GSAS) software. The micro structural features of the micro grains in sintered pellets were studied by Scanning Electron Microscopy (SEM, JEOL JSM-6700) and lattice fringes were observed by Transmission Electron microscopy (TEM, JEM-2010–200 kV). Raman spectra is measured using argon laser (532 nm) with a Kaiser holographic edge filter (JASCO Hololab 5000). Ferroelectric polarization was measured with a P-E loop tracer (Radiant Technologies, USA). For magnetic behavior of the samples were measured with a 5 T mini vibrating sample magnetometer (VSM) from Cryogenic Ltd. The magneto capacitance (MC) effect is measured with LCR meter (model HIOKI 3532–50, Japan) by applying a different magnetic field ranging from 0 Oe to 7 kOe.

## Results and discussion

### Structural analysis

**Figure 1:**
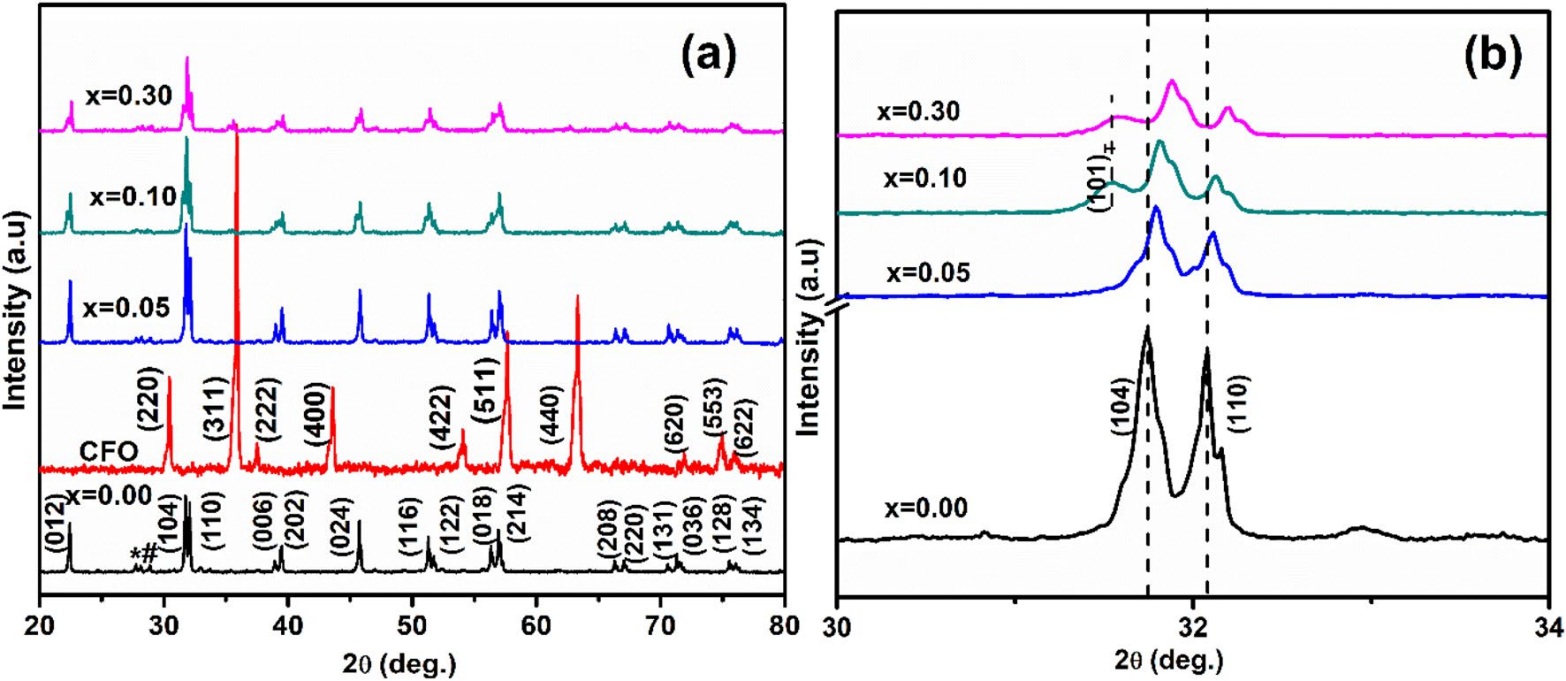
**(a)** X-ray diffraction patterns of (1 − x) BFO–xCFO (CFO, x = 0.00, 0.05, 0.10 and 0.30) ceramic powders calcined at 840 °C for 3 h (*Bi_24_Fe_2_O_39,_ #Bi_2_Fe_4_O_9_). **(b)** Expanded XRD patterns range between 2θ = 30°–34° and showing corresponding to tetragonal (101)_T_ and rhombohedral (104)/(110) planes.

XRD patterns of (1 − x) BFO-xCFO (CFO, x = 0.00, 0.05, 0.10 and 0.30) ceramics and BFO and CFO powders calcined at 840 °C for 3 h as shows in Fig. 1. The XRD pattern of BFO shows peaks corresponding to the BiFeO_3_ rhombohedral structure (*R3c,* JCPDS 71-2494) and the impurity phases of *Bi_24_Fe_2_O_39,_ #Bi_2_Fe_4_O_9_ which was formed due to the kinetics of formation^15,16^. The XRD pattern of CFO indicated the cubic inverse spinal structure with space group of *Fd3m* matched well with JCPDS Card No. 20-0169. As demonstrated in Fig. 1b, the 0.95BFO-0.05CFO reserved the rhombohedral structure for relatively lower CFO concentration. However, with increasing CFO concentration to x = 0.1 and x = 0.3, new peak has appeared at 2θ = 31.5° which suggests that this new peak is corresponding to tetragonal structure with a space group of (*P4mm)*^17–20^. In addition to that, the X-ray diffraction peaks are shifted to the higher angle with increasing CFO concentration, which indicates the shrinkage of the BFO unit cell due to the substitution of Co^3+^ (0.685 Å) cation at the site of Fe^3+^ (0.785 Å).

**Figure 2:**
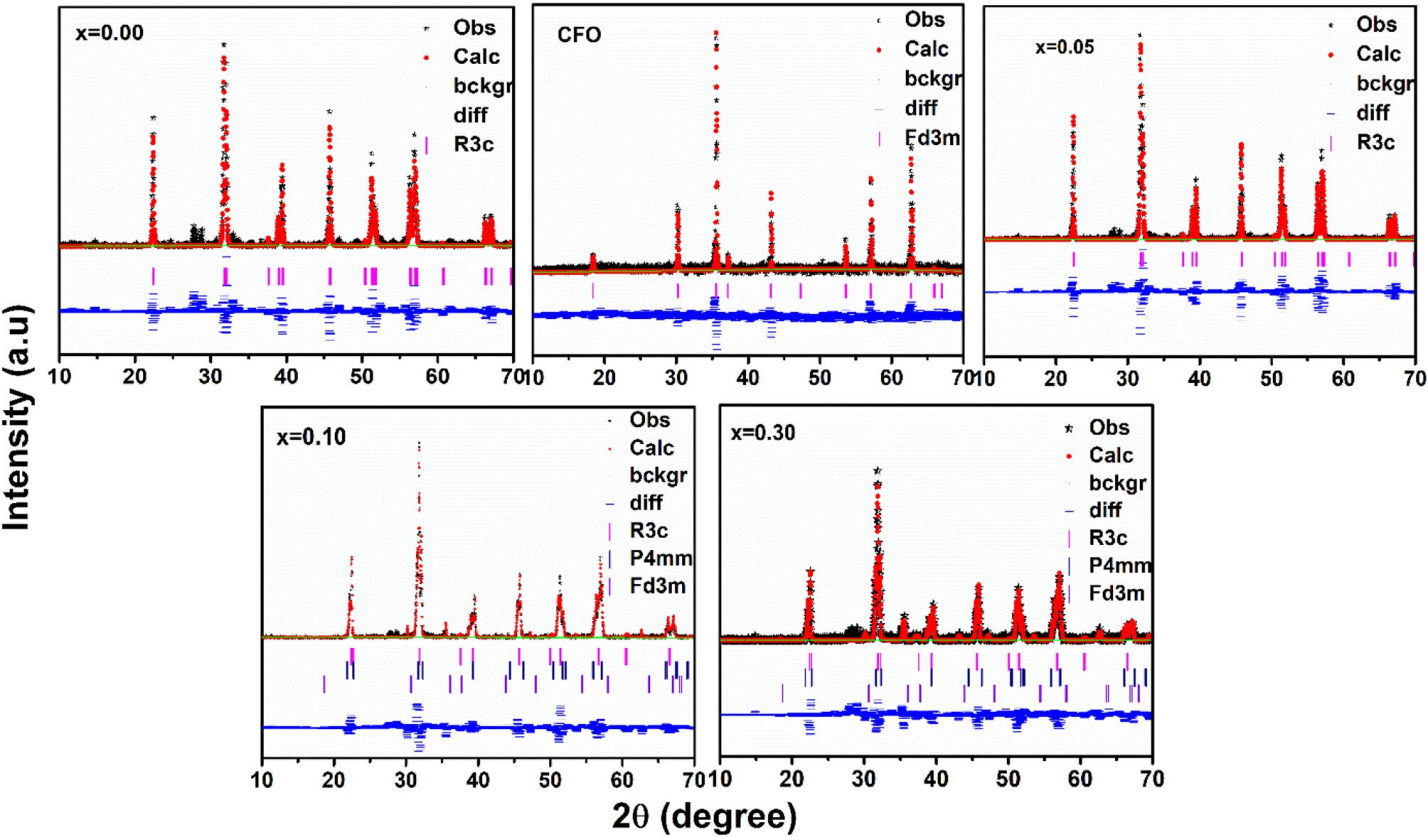
Rietveld refinement profiles of the X-ray diffraction data for (1 − x) BFO–xCFO (CFO, x = 0.00, 0.05, 0.10 and 0.30) ceramic samples. Observed (Obs), calculated (Calc), and difference (diff). The Bragg reflections are denoted by vertical sticks and solid lines are fitted in background.

According to literature reports, the splitting of the (101) and the (101)/(104) reflections of the coexistence of rhombohedral (*R3c*) and tetragonal (*P4mm*) phases into triplets is usually taken as an evidence for a tetragonally distorted phase. Singh et al.^17^, reported that BiFe_1-x_Co_x_O_3_ transforms from rhombohedral R (*R3c*) to tetragonal T (*P4mm*) crystal structures as the cobalt concentration increases and both phases (R and T) coexist for Co content between x = 0.10 and 0.20. These morphotropic phase boundary (MPB) samples may potentially display the magneto capacitance (MC) effect with a variance in the macroscopic magnetization because only rhombohedral domains exist for Co concentration below x = 0.20. Azuma et al.^18^ studied the (1-x) BiCoO_3_–xBiFiO_3_ composite system consisting tetragonal (*P4mm*) to rhombohedral (*R3c*) phase boundary between x = 0.6 and x = 0.8 at room temperature. Nakamura et al.^19^ reported the (1 − x) BiFeO_3_–xBiCoO_3_ thin films which shows the rhombohedral and tetragonal phases coexisting in the range of x = 0.075 and x = 0.15. Oka et al.^20^ reported tetragonal (*P4mm*) to rhombohedral (*R3c*) phase transition at high temperature in BiCo_1−x_Fe_x_O_3_ (x = 0.7) and a monoclinic phase is found at room temperature in the vicinity of this composition x = 0.8. Based on these research lines, we attempted three different structural models of *R3c, P4mm, Fd3m* to fit the XRD patterns of the prepared (1 − x) BFO–xCFO composite powders with concentration of CFO up to x = 0.30 sample. The Rietveld refinement results carried out using X-ray diffraction data for all the samples are shown in Fig. 2. The refinement is revealed that using the rhombohedral crystal symmetry (*R3c*) for BFO and (1 − x) BFO–xCFO (x = 0.05) ceramic were well matched with experimental data. The crystal structural information obtained from Rietveld refinement results are summarized in Table 1.

**Table 1:** Relevant parameters obtained from Rietveld refinement of XRD pattern for [(1 − x) BFO–x CFO (CFO, x = 0.00, 0.05, 0.10 and 0.30)] samples.

1 − x BFO–xCFO (CFO, x = 0.00, 0.05, 0.10 and 0.30)	Crystal structure	Lattice parameters (Ǻ) Volume (Å)	Atomic positions	R factors (%)
x = 0.00	Rhombohedral (*R3c*)	a = 5.5830 c = 13.874 V = 374.536	Bi 0 0 0 0 Fe 0 0 0 0.220 O 0.449 0.0181 0.9518	_W_R_p_ = 22.5 R_p_ = 15.70 χ^2^ = 2.09
CFO	simple cubic (*Fd3m*)	a = 8.388 V = 590.311	Co 0.125 0.125 0.125 Fe1 0.125 0.125 0.125 Fe2 0.500 0.500 0.500 O 0.2566 0.256 0.256	_W_R_p_ = 25.4 R_p_ = 18.10 χ^2^ = 3.32
x = 0.05	Rhombohedral (*R3c*)	a = 5.578 c = 13.861 V = 373.541	Bi 0 0 0.446 Fe/Co 0 0 0.0181 O 0 0.2202 0.9518	_W_R_p_ = 22.7 R_p_ = 16.3 χ^2^ = 2.47
x = 0.10	Rhombohedral (*R3c*) 35.01% Tetragonal (*P4mm*) 43.51% simple cubic (*Fd3m*) 21.48%	a = 5.5808, c = 13.880 V = 374.22 a = 3. 913 c = 4.14 V = 63.418 a = 8.384 V = 589.367	Bi 0 0 0 Fe/Co 0 0 0.220 O 0.446 0.0181 0.951 Bi 0 0 0 Fe/Co 0.500 0.500 0.1140 O 0.500 0.00 0.6170 Co 0.125 0.125 0.125 Fe1 0.125 0.125 0.125 Fe2 0.500 0.500 0.500 O 0.2566 0.256 0.256	_W_R_p_ = 21.6 R_p_ = 15.0 χ^2^ = 2.14
x = 0.30	Rhombohedral (*R3c*) 25.75% Tetragonal (*P4mm*) 44.12% simple cubic (*Fd3m*) 30.13%	a = 5.5901, c = 13.839 V = 375.788 a = 3.912 c = 4.130 V = 63.280 a = 8.389 V = 587.56	Bi 0 0 0 Fe/Co 0 0 0.220 O 0.446 0.0181 0.951 Bi 0 0 0 Fe/Co 0.500 0.500 0.114 O 0.500 0.00 0.5390 Co 0.125 0.125 0.125 Fe1 0.125 0.125 0.125 Fe2 0.500 0.500 0.500 O 0.256 0.256 0.256	_W_R_p_ = 25.3 R_p_ = 18.45 χ^2^ = 2.61

**Figure 3:**
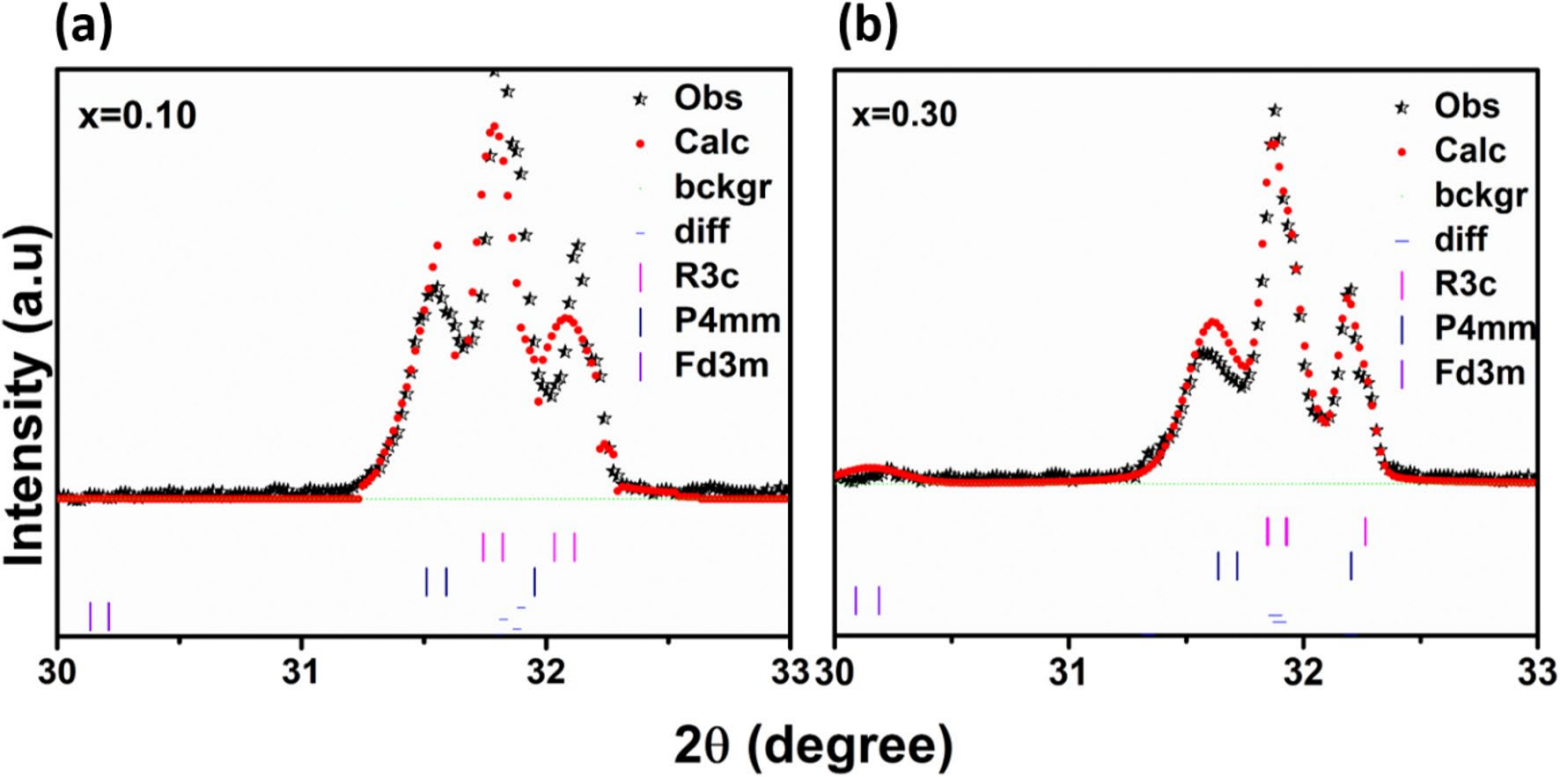
The refined profiles using the **(a,b)** *R3c* + *P4mm* + *Fd3m* symmetries across the (110)_T_ and (104)/(110)_R_ reflections (referring to tetragonal (T) with rhombohedral (R) indices) are revealed to show the better agreement of the *P4mm* phase.

As shown in the Fig. 3a,b, the Bragg profiles could be fitted reasonably well with the tetragonal structural model (101), along with rhombohedral structure (101)/(104), which clearly confirms the coexistence of both structures in the composites. From the refinement results, we have observed the slightly variation in lattice parameter, volumes and phase fractions according to CFO concentration increasing into BFO. It has been observed structural phase transformation from rhombohedral (*R3c*) to other structural phases started at x = 0.10, which can be considered as a morphotropic phase boundary.

### Microstructural analysis

**Figure 4:**
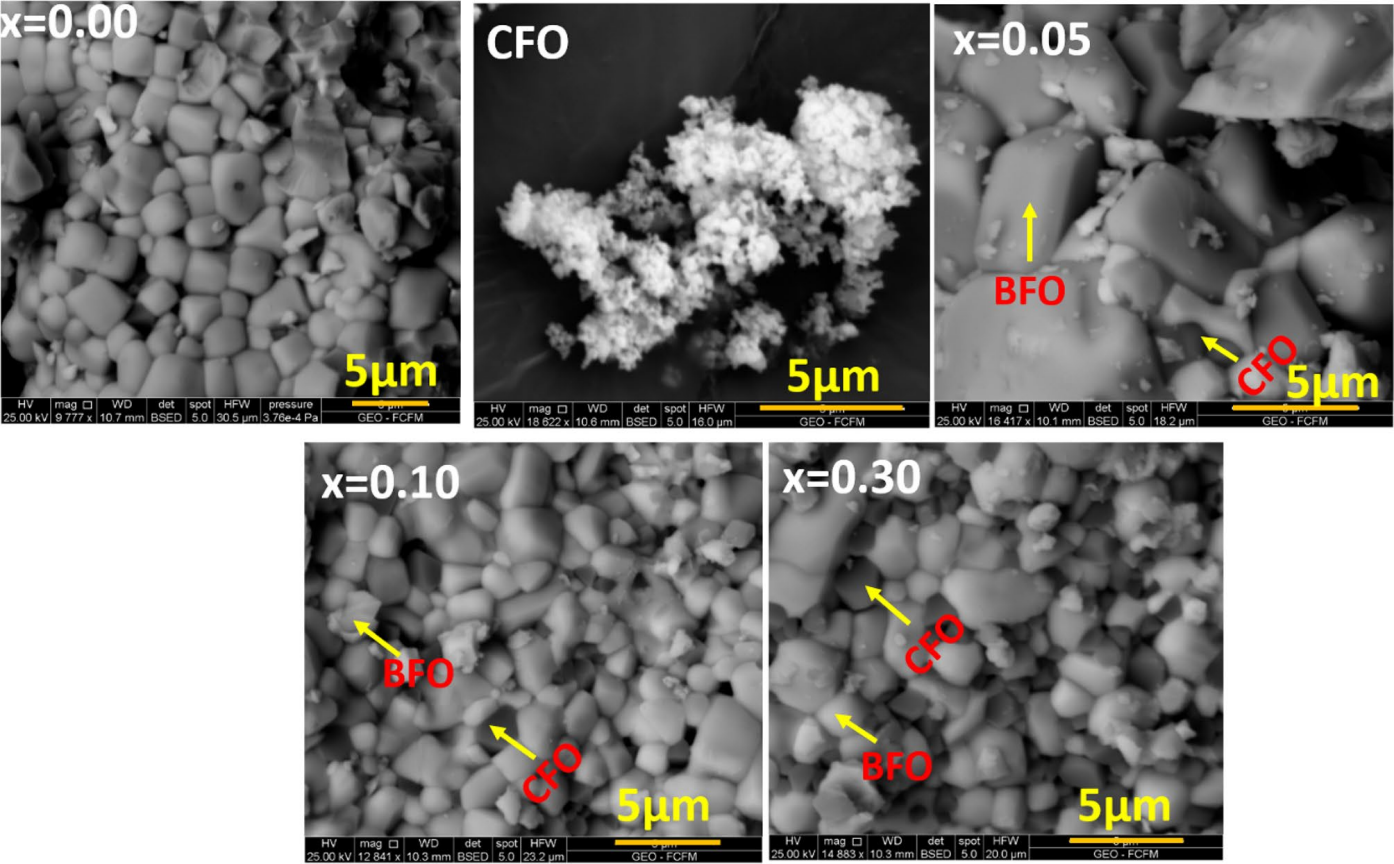
SEM images of (1 − x) BFO–xCFO (CFO, x = 0.00, 0.05, 0.10 and 0.30) ceramic pellets sintered at 980 °C for 5 h.

SEM images of (1 − x)BFO–xCFO (CFO, x = 0.00, 0.05, 0.10 and 0.30) ceramics, BFO and CFO pellets sintered at 980 °C for 5 h are shown in Fig. 4. As it can be seen, semi-rectangular agglomerated BFO particles with average grain size of 1.6 µm are observed in BFO sample. In composite powders, CFO particles are distinguishable and composite nature of their microstructure is evident. However, the white grains are belonging to BFO and dark grains are belong to CFO. The average grain size is calculated for (1 − x) BFO–xCFO (x = 0.05, 0.10 and 0.30) composites and the samples are 2.0, 1.36 and 1.19 µm, respectively. This decreasing trend with the increasing of CFO concentration in the composites might be due to the role of the impurity ions which is to suppress the growth of the grains in the heterogeneous nucleation sites^21^.

**Figure 5:**
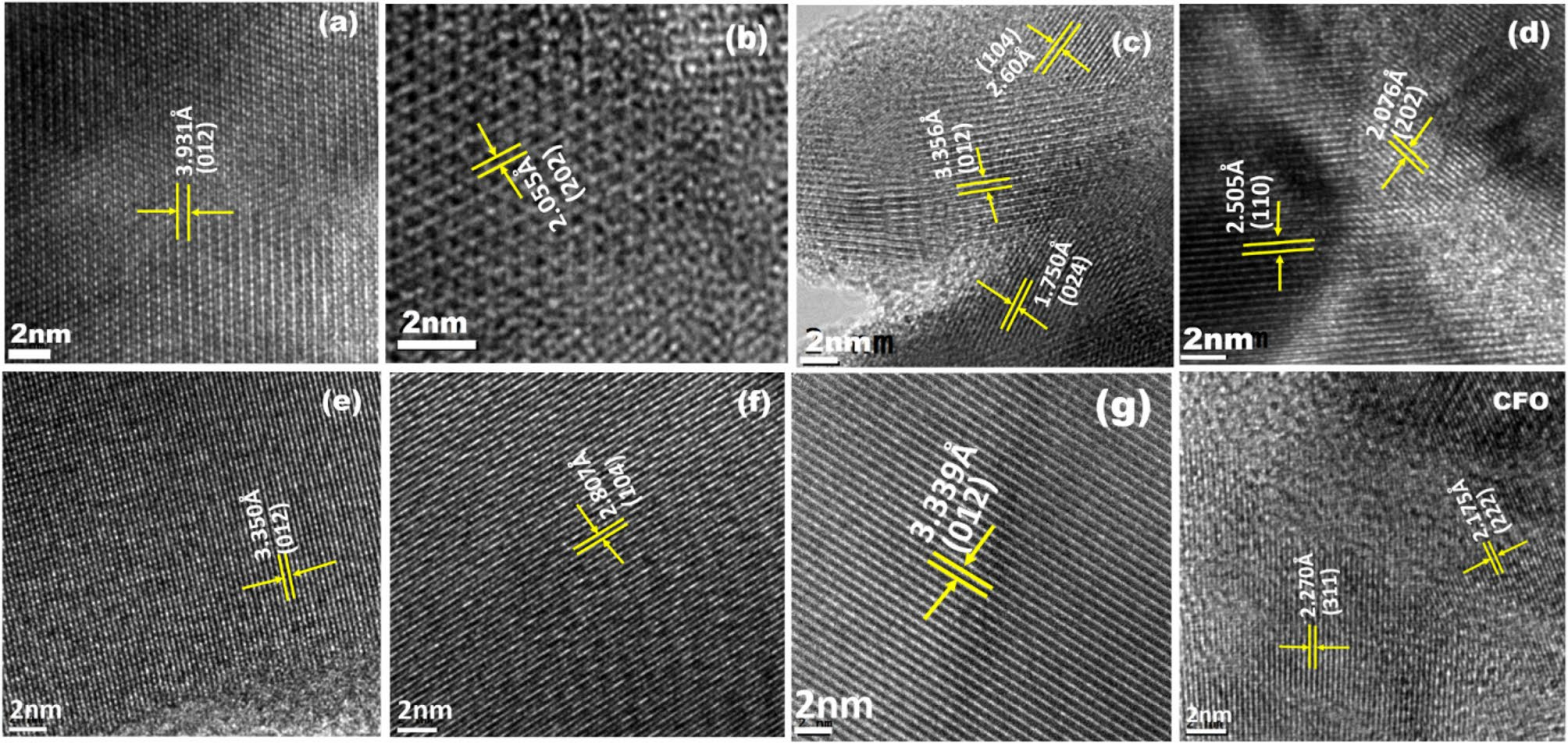
High resolution transmission electron microscope image of (1 − x) BFO–xCFO, [x = 0.00 (**(a,b)**, 0.05 **(c,d)**, 0.10 **(e,f)**, 0.30 **(g)**, CFO)] samples.

HRTEM images of calcined BFO–CFO powders consist of clear internal crystal lattice structures, which confirm their high crystallinity as shows in Fig. 5. As depicted in Fig. 5, the lattice fringes measured with lattice spacing by using Image J software and are well matched with (012), (104) and (012) of BFO phase corresponding to (1 − x) BFO–xCFO (x = 0.05, 0.10 and 0.30) sample respectively.

### Raman spectra

**Figure 6:**
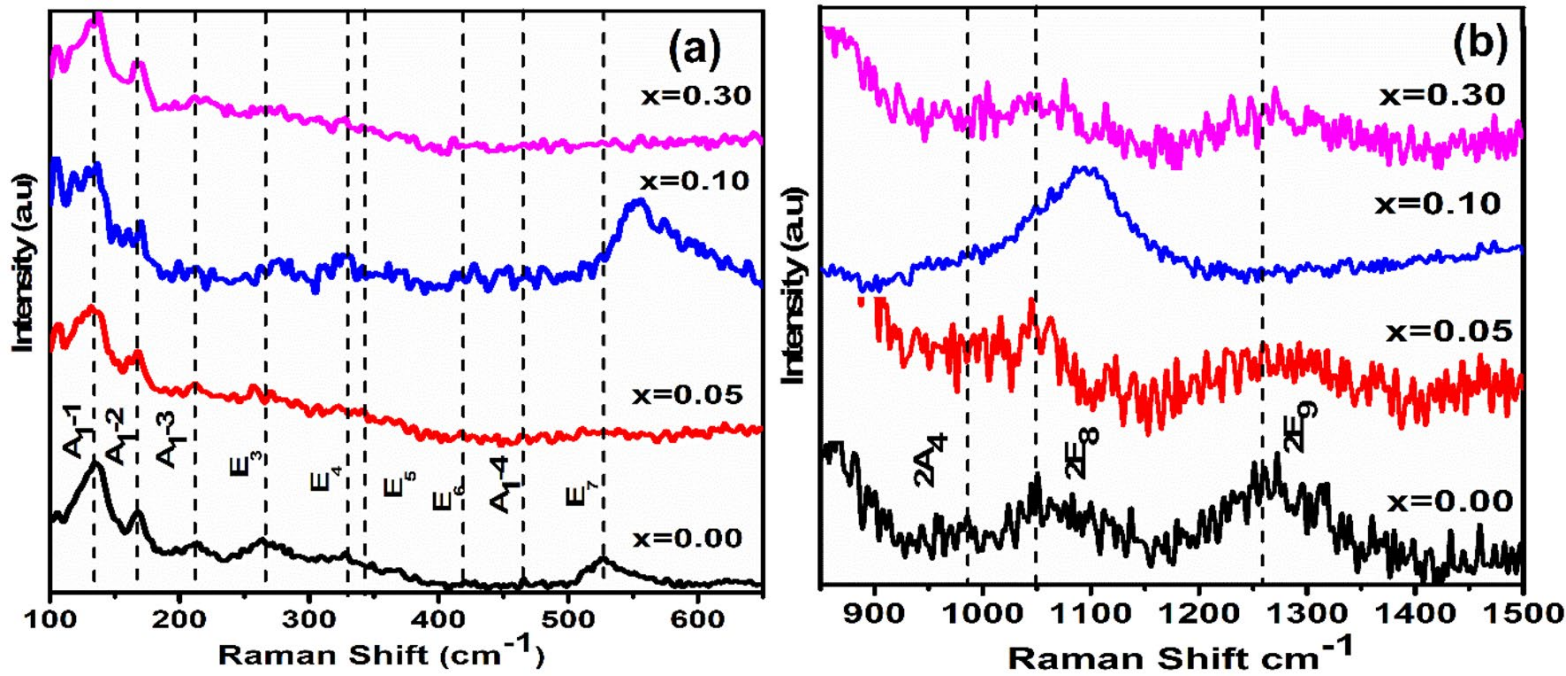
**(a)** Raman spectra in the range between 100 and 650 cm^−1^ at room temperature and **(b)** two phonon modes in the range of 850–1500 cm^−1^ of (1 − x) BFO–x CFO (x = 0.00, 0.05, 0.10 and 0.30).

Raman spectra measured for (1 − x) BFO–xCFO (x = 0.00, 0.05, 0.10 and 0.30) ceramics and BFO calcined powders in the range of wave number from 100 to 650 cm^−1^ at room temperature are shown in Fig. 6a. According to the group theory, rhombohedrally distorted crystal structure with space group of *R3c* having 13 Raman-active modes are predicted, which is given as formula^22^:

$$\Gamma_{{{\text{Raman}}}} = {\text{ 4A}}_{{1}} + {\text{ 9E}}$$

Here, the A_1_ and E modes are representing transverse and the longitudinal optical modes, respectively as Raman active modes. For BFO samples have been observed 4A_1_ and 5E modes are 134, 166, 210, 265, 326, 343, 416, 463, and 525 cm^-1^, which matches with the previous literature reports^23^. the low-frequency range Raman modes are mostly associated with the Bi-O vibration and the higher frequency modes are associated with Fe–O vibrations^24^. Thus, the origin of ferroelectricity is due to the stereochemical activity of the Bi-6s^2^ lone pair electron and changes in the BFO covalent bonds. In the present work, there are six characteristics of Raman modes (i.e., E1, A_1_-1, A_1_-2, A_1_-3, A_1_-4 and E_2_) these are assisted in inducing ferroelectricity of BFO. For 0.95BFO-0.05CFO sample, 4A_1_ and 5E Raman modes were observed at 133, 168, 211, 265, 330, 342, 416, 463 and 528 cm^−1^, while E_7_ Raman mode almost vanished. For 0.9BFO-0.1CFO sample, Raman modes were observed at 170, 275, 426, and 550 cm^−1^ modes that were slightly shifted to higher wavenumber, which may be attributed to the lower atomic weight of Fe^3+^ (55.845 g/mol) than that of Co^3+^ (58.93 g/mol) and E_7_ modes appeared with high intensity, this may be due to the introduced new crystal structure along with rhombohedral *R3c* or suggest a more complex signature with overlapping and super imposing features of BFO–CFO^25,26^. Further increasing of CFO concentration to x = 0.3 in (1 − x) BFO–xCFO resulted to almost disappearance of the Raman modes, which indicates a reduce in the stereochemical activity of the Bi (6s^2^) lone pair electron, which induces a sudden change in Bi–O covalent bonds possibly to the structural transformation from the rhombohedral to tetragonal crystal structure. Raman studies declare structural changes from rhombohedral to tetragonal for [(1 − x) BFO–xCFO (x = 0.10 and 0.30)] samples, which confirms the Rietveld refinement analysis of X-ray diffraction data.

### Two phonons scattering

The two phonon Raman scattering for BFO and (1 − x) BFO–xCFO (x = 0.00, 0.05, 0.10 and 0.30) ceramic powders in higher order and the frequency range between 850 and 1500 cm^−1^ are shown in Fig. 6b. Generally, the origin of the high-frequency modes in the Raman spectra is attributed to electronic Raman scattering or the high-order phonon scattering. BFO shows three higher order Raman modes, namely, 2A_4_ (LO), 2E_8_ (TO), and 2E_9_ (TO) at 960, 1099, and 1261 cm^−1^, respectively, which are overtones of the first-order A_4_, E_8_, and E_9_ phonon modes^9^. For (1 − x) BFO-xCFO (x = 0.10) sample, 2A_4_ and 2E_9_ modes are completely vanished and 2E_8_ Raman modes are shifted towards to the higher wavenumber with higher intense, which is occurred near MPB in the region. These changes can be attributed to the structural transformation from rhombohedral to tetragonal structure^9^. For (1 − x) BFO–xCFO (x = 0.30) samples, the some of the two phonons modes are vanished, which may be indicated as a weakening of the ferroelectricity at BFO. 2E_8_ (TO), and 2E_9_ (TO) modes are attributed to Fe–O1 and Fe–O2 bonding, where O1 are axial ions and O2 are equatorial ions^23^. As a results of Raman spectroscopy, it can be understood from structural transition of rhombohedral to tetragonal at (1 − x) BFO–xCFO (x = 0.10 and 0.30) samples are evidenced from the X-ray diffraction data.

### Leakage current measurement

**Figure 7:**
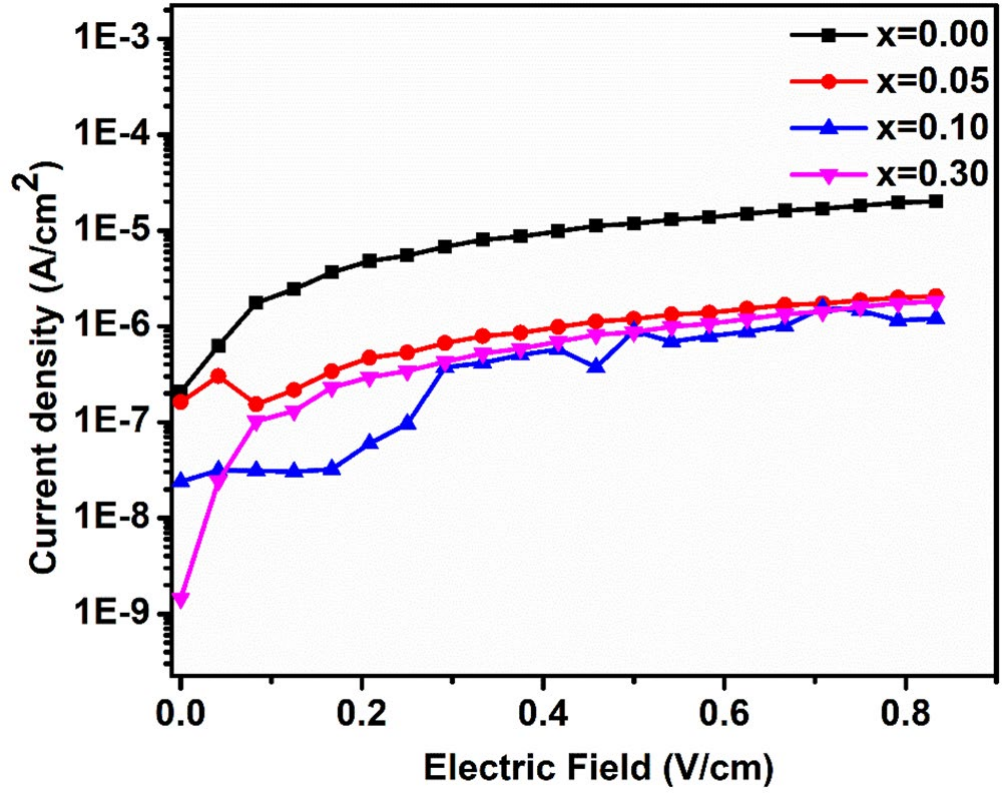
Leakage current density measured in (1 − x) BFO–xCFO (x = 0.00, 0.05, 0.10 and 0.30) ceramics.

The measured leakage current density of (1 − x)BFO–xCFO (x = 0.00, 0.05, 0.10 and 0.30) ceramic samples as a function of applied different positive electric fields (E) are shown in Fig. 7. As it can be seen, when the applied electric filed increases, the leakage current density increases for all the samples. It is noticed that the pure BFO has higher leakage current density, which may be by the presence of charged oxygen vacancies due to the reduction of Fe^3+^ to Fe^2+^ in BFO^27,28^. The leakage current density increased with increasing CFO concentration except 0.9BFO–0.1CFO sample, which showed the lowest leakage current density of 1.25 × 10^–6^ A/cm^2^ at MPB.

### Ferroelectric measurement

**Figure 8:**
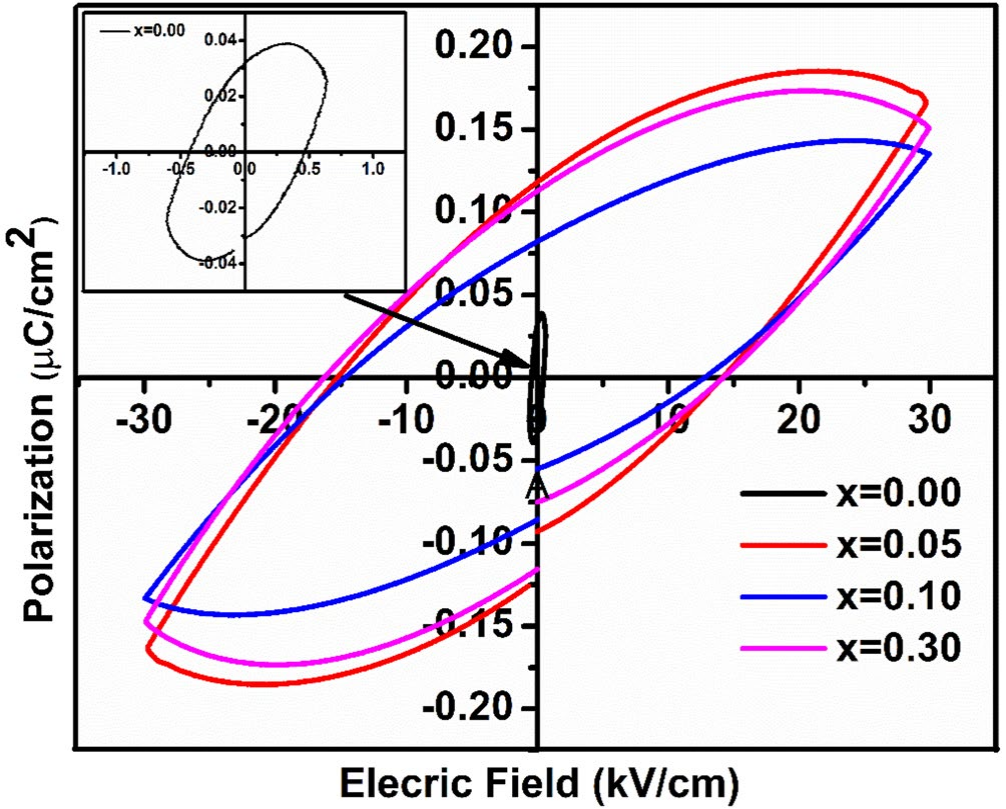
Polarization versus electric field (P–E) hysteresis loops of (1 − x) BFO–xCFO (x = 0.00, 0.05, 0.10 and 0.30) ceramic samples**.**

Ferroelectric behaviour of BFO and (1 − x) BFO–xCFO (x = 0.00, 0.05, 0.10 and 0.30) sintered discs were characterized at room temperature in terms of polarization versus electric field (P–E) hysteresis loops as shown in Fig. 8. The polarization values measured from the ferroelectric hysteresis loop such as Remnant polarization (P_r_) and saturated polarization (P_s_) are listed in the Table 2. Rounded ferroelectric hysteresis loops for BFO, which indicates their ferroelectric characteristic and improper hysteresis loop behaviour have been observed. These kinds of ferroelectric behaviours are may be due to the higher leakage current density in the BFO^10,29^. As it can be seen at MPB region, the polarization values are high at (1 − x) BFO–xCFO (x = 0.05 and 0.30) with applied electric field 30 (kV/cm) to the samples, then compared to x = 0.10 sample. These changes in the polarization of all the ceramic samples may be attributed to the structural transition from rhombohedral to tetragonal, which is in good agreement with X-ray diffraction and Raman studies.

**Table 2:** Calculated recoverable energy density (W_R_), energy loss density (W_L_), efficiency of energy storage density (ƞ), coercive field (E_c_), maximum polarization (P_max_), remnant polarization (P_r_), remnant magnetization (M_r_), saturation magnetization (M_s_) for [(1 − x) BFO–xCFO (x = 0.00, 0.05, 0.10 and 0.30)] ceramic samples.

Samples (1 − x) BFO–xCFO	E_c_ (kV/cm)	P_r_ (μC/cm^2^)	P_max_ (μC/cm^2^)	W_L_ (J/cm^3^)	W_R_ (J/cm^3^)	ɳ%	M_r_ (emu/g)	M_s_ (emu/g)
BFO	0.476	0.0318	0.0384	0.0126 × 10^–1^	0.0066 × 10^–1^	34.37	4.6 × 10^–6^	6.7 × 10^–6^
x = 0.05	14.1	0.118	0.160	1.14 × 10^–1^	1.26 × 10^–1^	52.5	2.62	11.51
x = 0.10	12.7	0.088	0.134	0.63 × 10^–1^	1.38 × 10^–1^	68.65	9.99	39.10
x = 0.30	14.2	0.111	0.148	1.11 × 10^–1^	1.11 × 10^–1^	50	34.50	139.104

To investigate further the energy storage density of ceramic samples, the P_r_, P_max_ and E_c_ values were used for calculation of recoverable energy density (W_R_) and total energy density (W_T_) by using Eqs. (6) and (7), respectively.

$${\text{W}}_{{\text{R}}} = \mathop \smallint \limits_{Pr}^{Pmax} Edp$$

$${\text{W}}_{{\text{T}}} = \frac{1}{2}Pmax E$$

where, P_max_ is a maximum polarization and P_r_ is the remanence polarization.

The efficiency of energy density calculated by using Eq. (8)

$$\eta \% = \frac{{W_{R} }}{{W_{R} + W_{L} }} \times 100$$

where ‘η’ represents efficiency of energy density and the energy loss density (W_L_) obtained by W_L_ = W_T_ + W_R_.

The enhanced energy storage density was observed for BFO–CFO samples, which may be due to the improved dielectric breakdown strength E_b_, compared with pure BFO. In addition, the introduction of CFO leads to reduced grain size (on microscale), which is responsible for the enhanced dielectric breakdown strength E_b_^30^. As reported in Table 2, the recoverable energy density increased with increasing in CFO concentration upto x = 0.10, then decreased for higher CFO concentrations of x = 0.30. Hence, the maximum recoverable energy density of 0.138 J/cm^3^ and efficiency of energy density (ɳ = 68.65%) was achieved at 0.90BFO-0.10CFO sample near to MPB, which is about twenty times higher than that of pure BFO (0.0066 × 10^–1^ J/cm^3^). A comparison of ferroelectric and energy storage density properties among BFO based ceramic systems are listed in the Table 3. It can be seen that the recoverable energy storage density and efficiency values are relatively matching with reported literature^10,31–33^. The maximum efficiency of energy density is observed for x = 0.10, which indicates that the higher electric field is favourable for energy storage applications.

**Table 3:** Comparison of ferroelectric and energy storage properties of recently reported BFO based ceramics samples.

Samples	Applied electric field (kV/cm)	P_max_ (µC/cm^2^)	P_r_ (µC/cm^2^)	Energy storage density, W_rec_ (J/cm^3^)	Efficiency Ƞ (%)	Ref
0.70BiFeO_0.95_Mn_0.05_O_3_–0.30CoFe_2_O_4_	–	1.7 × 10^–3^	6.8 × 10^–4^	0.030	59.94	^10^
0.70(0.70BiFeO_3_–0.30BaTiO_3_)–0.30CoFe_2_O_4_	12	3.59	2.97	0.037	89.40	^31^
0.70(0.65BiFeO_3_–0.30BaTiO_3_)–0.30Nb_2_O_5_	90	25	5.3	0.71	–	^32^
0.07BiFeO_3_–0.33BaTiO_3_–0.60Ba(Mg_1/3_Nb_2/3_)O_3_	12.5	38	5.7	1.56	75	^33^
0.90BiFeO_3_–0.10CoFe_2_O_4_	30	0.134	0.088	0.138	68.65	Present study

### Magnetic measurement

**Figure 9:**
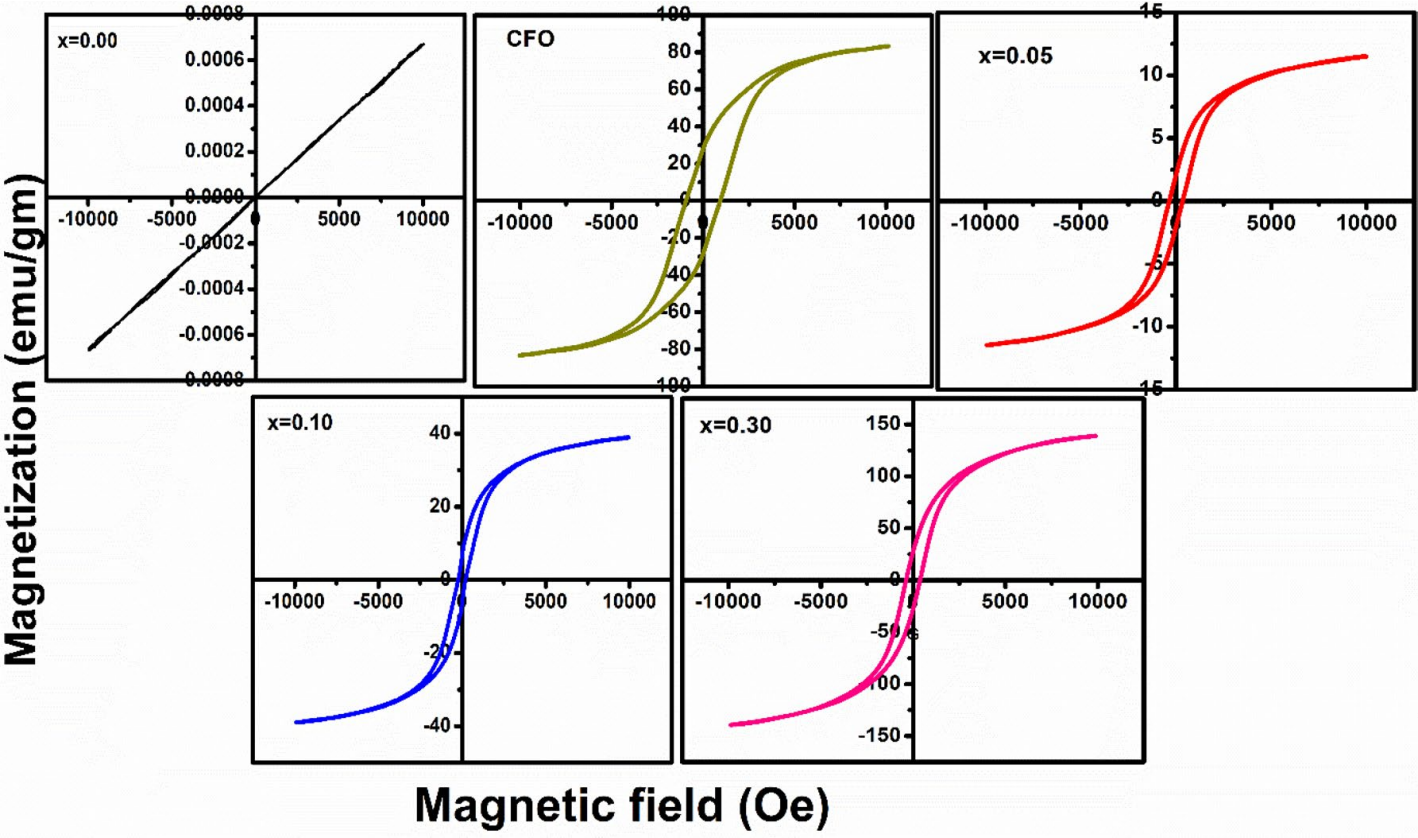
Magnetization (M) versus magnetic field (H) hysteresis loops of (1 − x) BFO–x CFO (CFO, x = 0.00, 0.05, 0.10 and 0.30) samples at room temperature.

As shown in Fig. 9, magnetic properties of prepared composite powders were obtained by studying their magnetic hysteresis loops and compared with those of BFO and CFO powders. The BFO sample shows a linear M-H loop, which indicates its antiferromagnetic behaviour at room temperature due to the G-type of modulated spiral spin structure with long periodicity of 62 nm in the unit cell at bulk samples^34^. CFO sample shows proper.

M-H hysteresis loop, which show its ferromagnetic behaviour^35^. The enhancement of magnetization of BFO-CFO samples with increasing CFO concentration can be seen as they show a well-saturated hysteresis loop so that all the BFO-CFO samples will show ferromagnetic behaviour with a large saturation magnetization. CFO exhibits a ferromagnetic behaviour with saturation magnetization (M_s_) and remnant magnetization such as (M_r_) of 83.17 emu/gm and 30.03 emu/gm. The obtained huge saturation magnetization (M_s_) was achieved around 139.10 emu/g for x = 0.30. These magnetization values are higher than that of BFO, CFO samples which were reported earlier as nano particle and bulk ceramic materials^36,37^. The coercivity (H_c_) increased sharply with increasing CFO concentration up to x = 0.30 and these results are suggesting the suitable candidates for spintronic applications. The values of M_s_ and M_r_ for all compositions are obtained from the hysteresis loops and are summarised in Table 2. For all the BFO–CFO samples, the enhancement of ferromagnetism can be attributed to the highly magnetic nature of CFO phase in the composite. There are some reason for enhancement of magnetisation of (1 − x) BFO–x CFO (x = 0.05, 0.10 and 0.30) samples, which may be due to some factors such as; (i) BFO has weak magnetization, it is a canted antiferromagnet, the spin orientations of the BFO grains are changed by the strain in the grain boundaries between the BFO and CFO in the composite samples and (ii) By adding CFO ions into BFO, induce a structural distortion near MPB (from rhombohedral to tetragonal phase transition), because the spin cycloid structure becomes destroyed with the formation of homogeneous spin structure. (iii) The increase of the magnetization of the composite is caused by the magnetic interaction between two compounds, i.e. the compounds are exchange coupled. Thus, the magnetic properties of the BFO can be improved by making composite with CFO^38^.

**Figure 10:**
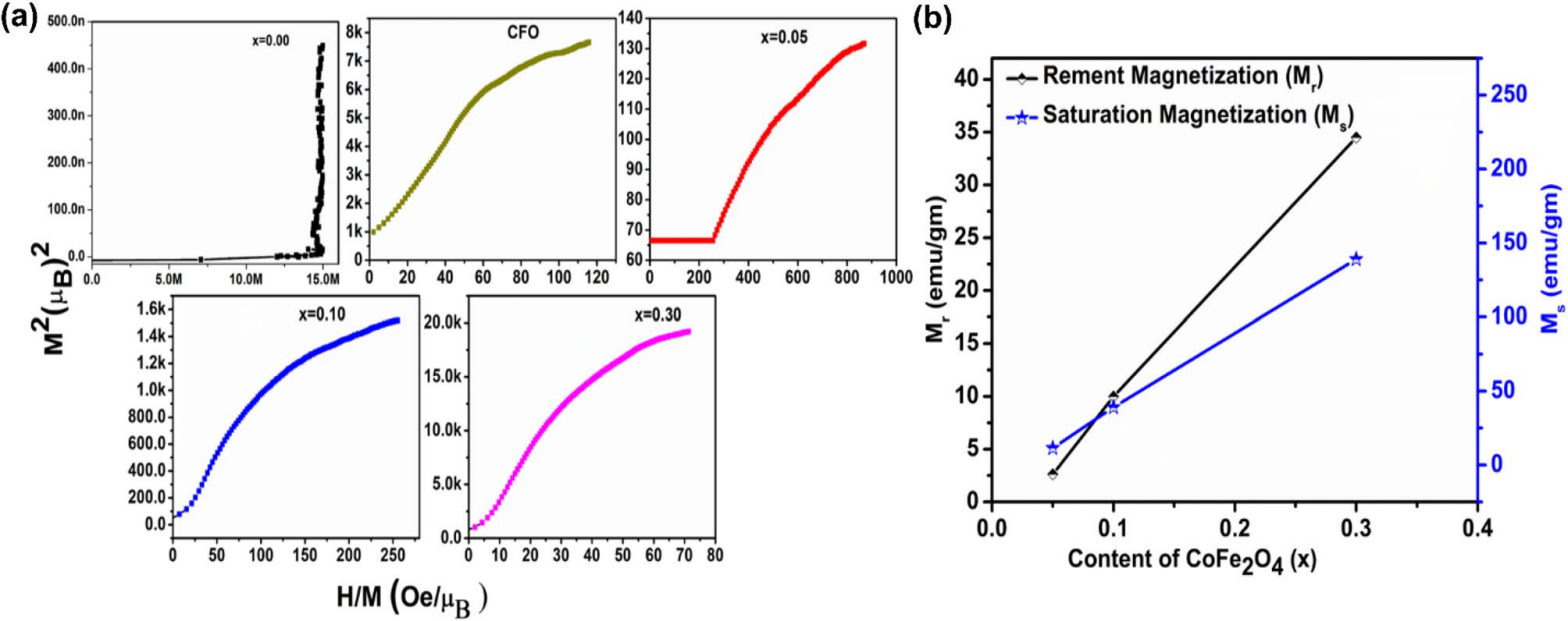
**(a)** Arrott–Belov–Kouvel (ABK) plots of (1 − x)BFO–xCFO (CFO, x = 0.00, 0.05, 0.10 and 0.30) samples at room temperature, **(b)** remnant magnetization and saturation magnetization versus CFO concentration.

In order to confirm the magnetic behaviour of (1 − x) BFO–xCFO (CFO, x = 0.00, 0.05, 0.10 and 0.30) composite samples, Arrott– Belov–Kouvel (ABK), magnetic field as a function of magnetization were plotted and compared with that of BFO sample as shown in Fig. 10a. For BFO sample, ABK plot shows a concave nature without any spontaneous magnetization at H = 0, which indicates its antiferromagnetic behaviour. (1 − x)BFO–x CFO (x = 0.05, 0.10 and 0.30) samples show some spontaneous magnetization values even at H = 0 as ABK plots exhibit a convex curve, which confirm their ferromagnetic behaviour at room temperature. The values of saturation magnetisation (M_s_) linearly increased with increasing in CFO concentration and the values of remnant magnetization (M_r_) increased with CFO concentration are shown in Fig. 10b.

### Magneto-capacitance measurement

**Figure 11:**
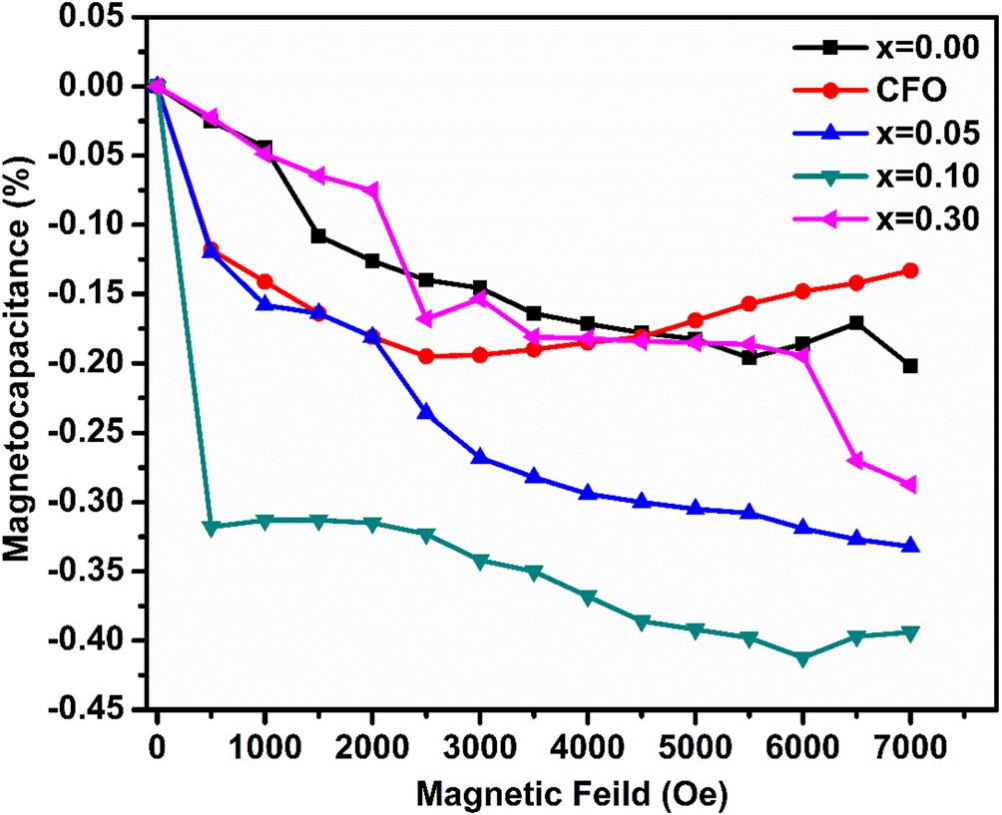
Magneto-capacitance versus magnetic field measurement of (1 − x)BFO–xCFO (CFO, x = 0.00, 0.05, 0.10 and 0.30) samples at room temperature.

In order to explain the magneto capacitance effect of (1 − x) BFO–xCFO (CFO, x = 0.00, 0.05, 0.10 and 0.30)] ceramic samples, the variation of the capacitance measured with respect to the applied magnetic field at room temperature and compared with those of BFO and CFO samples as shown in Fig. 11. In general, it is known that all ferroelectrics which are piezoelectric and piezoelectric crystals become electrically polarized under applied stress. Hence, the mechanism of magneto electric effect due to the strain will be induced by applied external magnetic field to the multiferroic materials and it becomes strained. The strain generates a stress in the material, which is generated as an electric field on the ferroelectric domain, thus modifying the electric polarization and dielectric constant of the samples. The coexistence of the ferromagnetic CFO and ferroelectric BFO phases in the present composites give rise to a magneto-electric (ME) effect. This ME effect is calculated in terms of magneto-capacitance effect by using eqn.:

9 $$\mathrm{Magneto}-\mathrm{Capacitance}=\frac{Cp\left(H\right)-Cp \left(0\right)}{Cp\left(0\right)}100 (\%)$$

where C_P_(0) and C_P_(H) are the capacitance in zero and H magnetic fields, respectively.

It is clear from Eq. (9) that magnetoelectric coefficient depends on dielectric constant as well as magnetic moment of the sample. In the present work, it is found that the magneto capacitance effect increases with increase in applied magnetic field to the (1-x) BFO-x CFO (CFO, x = 0.00, 0.05, 0.10and 0.30) samples. BFO shows the weak magnetic response with applied magnetic field because of its antiferromagnetic nature with poor ferroelectricity at room temperature. It has been observed that the low magnetic response with applied magnetic field for CFO sample is due to the absent of ferroelectric with high magnetic moment. However, (1-x) BFO-x CFO (x = 0.05, 0.10 and 0.30) samples show a good magnetic response with applied magnetic field. The magneto capacitance effect increases with increasing CFO concentration up to x = 0.10 at near MPB, then decreases in composites with higher concentrations of x = 0.03. This indicates an improvement of magneto capacitance effect in BFO after formation of the nanocomposite. Magneto capacitance effect in a material generally originates from both intrinsic and extrinsic effects^39^. Hence, the magneto electric coupling which is caused by the magnetostriction of the CFO phase, which in turn enhancing the ME coupling in the magnetostriction of ferromagnetic domain from CFO and ferroelectric domain from BFO composites through stress mediated magnetic-mechanical–electrical transformed at the BFO–CFO composites interface^40^. Therefore, the presence of CFO phase in the composites enhances the magneto capacitance effect and makes the CFO–BFO composites as good candidates for the spintronic applications.

## Conclusions

The composition driven in structural transformation of the (1 − x) BFO–xCFO (CFO, x = 0.00, 0.05, 0.10 and 0.30) samples was investigated. (1 − x) BFO–xCFO (x = 0.10) shows a maximum energy storage density with strong magneto capacitance effect near to the MPB of tetragonal (*P4mm*) and the rhombohedral (*R3c*) phases. XRD shows composition-driven structural transformation from rhombohedral (*R3c*) to tetragonal with space group of (*P4mm*)*,* which is confirmed by Reitveld refinement analysis using X-ray diffraction data. SEM images show the rectangular shaped micro grains for BFO and BFO–CFO samples. From Raman spectra, two phonon scattering Raman modes were observed for the higher wavenumber and these spectrums were well supported for the structural transition of the crystal structure on the BFO–CFO. The polarization values are high at (1 − x) BFO–xCFO (x = 0.05 and 0.30) with applied electric field 30 (kV/cm) to the samples, then compared to x = 0.10 sample, which is described by changing polar symmetry of the crystal structure from rhombohedral to tetragonal. Here, we report a composites oxide that belongs to the (1 − x) BFO–xCFO (CFO, x = 0.00, 0.05, 0.10 and 0.30) binary system, chemically designed to present such morphotropic phase boundary with enhanced ferroelectricity, energy storage density and canted ferromagnetism, which show distinctive room-temperature magneto capacitance effect responses. These results might be useful for understanding and designing new technologies of these samples for spintronic applications.
